# CCR5 as a Prognostic Factor in Lower-Grade Glioma is Involved in the Remodeling of the Tumor Microenvironment

**DOI:** 10.3389/fgene.2022.874896

**Published:** 2022-07-05

**Authors:** Fang Wang, Zhennan Tao, Zhen Tian, Jiaqi Jin, Jiawei Dong, Yuxiang Dai, Wanli Yu, Bin Tang, Shaoshan Hu

**Affiliations:** ^1^ Department of Neurosurgery, The Second Affiliated Hospital of Harbin Medical University, Harbin, China; ^2^ Department of Neurosurgery, Emergency Medicine Center, Zhejiang Provincial People’s Hospital, Hangzhou Medical College, Hangzhou, China; ^3^ Department of Neurosurgery, the Affiliated Drum Tower Hospital, School of Medicine, Nanjing University, Nanjing, China; ^4^ Department of Minimally Invasive Interventional Oncology, Hubei Cancer Hospital, Tongji Medical College, Huazhong University of Science and Technology, Wuhan, China; ^5^ Department of Neurosurgery, The First Affiliated Hospital of Nanchang University, Nanchang, China

**Keywords:** tumor microenvironment, LGG, glioma, CCR5, immunological biomarker

## Abstract

**Background:** Lower-grade gliomas (LGGs) carry a high risk of malignant transformation, leading to severe neurologic deterioration and ultimately, death. The tumor microenvironment (TME) plays an essential role in tumor maintenance, progression, and immunotherapy resistance. Therefore, the LGG TME deserves comprehensive exploration for a novel therapeutic target.

**Methods:** The ESTIMATE algorithm was used to estimate infiltrating stromal and immune cells of LGG patients obtained from the Cancer Genome Atlas (TCGA) database. Kaplan–Meier analysis was performed to classify survival differences. TME-related differentially expressed genes were identified between the low- and high-immune/stromal groups. Hub genes were screened by constructing protein–protein interaction networks and performing the Cox regression analysis. Differential analysis, survival analysis, gene set enrichment analysis, and clinical relevance analysis specific to hub genes were evaluated by using the TCGA and the Chinese Glioma Genome Atlas datasets, and the results were validated by qRT-PCR, Western blotting, and immunohistochemistry in tissues from LGG patients.

**Results:** The immune and stromal components in TME were negatively related to patient prognosis. Differentially expressed genes sharing immune score and stromal score were mainly involved in the immune response. C-C chemokine receptor type 5 (CCR5), as only a hub gene, was significantly higher in LGG patients than normal patients and negatively correlated with the prognosis of patients. High-expression CCR5 was positively related to immune-related and tumor progression pathways. CCR5 protein expression was higher in LGG with isocitrate dehydrogenase wildtype. Validated results showed that CCR5 was upregulated in LGG tissues at mRNA and protein levels and could affect immune cell infiltration. These results suggested that CCR5 was a potential indicator for the status of TME.

**Conclusion:** Glioma cells remodel the immune microenvironment through the high expression of CCR5 and lead to a poor prognosis in patients with LGG. The inhibition of CCR5 may contribute to the efficacy of LGG immunotherapy.

## Introduction

Lower-grade glioma (LGG) is a subset of primary brain malignancy that causes severe neurological dysfunction and may progress toward glioblastoma (GBM) (Cuddapah et al., 2014; Lombardi et al., 2020). LGG has a better prognosis than GBM, with a median patient survival of 5.6–13.3 years (Louis et al., 2016). Modern comprehensive treatments are improving; however, LGG deterioration or recurrence still leads to an unsatisfactory outcome (Claus et al., 2015). Glioma recurrence relies on invasiveness and treatment resistance, which may partially ascribe to the peculiar tumor microenvironment (TME) (Hambardzumyan et al., 2016; Quail and Joyce 2017). Therefore, exploring the underlying mechanism of the interaction between glioma cells and the TME is helpful for developing effective therapeutic strategies.

The chemokine system has been widely demonstrated to be involved in the development of various tumors by promoting proliferation, metastasis, and angiogenesis, which can also modify the TME of tumor by recruiting leukocytes and enhancing angiogenesis (Balkwill 2012; Bachelerie et al., 2014; Korbecki et al., 2020). C-C motif chemokine receptor type 5 (CCR5), as a chemokine receptor, belongs to 7 transmembrane G-protein–coupled receptors and combines with different ligands to exert its effects. CCR5 is expressed on a broad array of tumors, and there is an increasing interest in developing CCR5 inhibitors as novel anticancer agents (Tan et al., 2009; Velasco-Velazquez et al., 2014; Jiao et al., 2019). A small number of studies have also reported the role of CCR5 in glioma. Some scholars found that CCR5 promoted invasion and metastasis of GBM through cell and animal experiments (Zhao et al., 2015; Novak et al., 2020). Another study revealed that pericytes augment glioblastoma cell resistance to temozolomide through CCL5–CCR5 paracrine signaling (Zhang et al., 2021). Interest is increasing on the role of CCR5 in glioma. However, since the current study focused solely on GBM, the roles of CCR5 in LGG still remain unclear. Not only that, there are no studies which used bioinformatics approaches to explore CCR5 in glioma.

In this study, with the bioinformatics and experimental analyses, we identified that CCR5 as a prognostic factor in lower-grade glioma is involved in the remodeling of the TME. The correlation between the expression of CCR5 with immune cell infiltration, immune-related signaling pathways, and biological processes was identified. This research provides new insights into the LGG-immune microenvironment, and our results offer further evidence for future clinical application of CCR5 inhibitors in glioma.

## Materials and Methods

### Data Collection and Processing

mRNA-seq datasets and clinical information of LGG cases were obtained from the Cancer Genome Atlas (TCGA) database (https://cancergenome.nih.gov/) and the Chinese Glioma Genome Atlas (CGGA) database (https://www.cgga.org.cn). mRNA-seq data of healthy samples were downloaded from the Genotype-Tissue Expression (GTEx) database (https://commonfund.nih.gov/GTEx/). The illuminaHumanv4. db R package was used to convert the probe IDs to gene symbols.

### Generation of Immune Score, Stromal Score and ESTIMATE Score

The R package “ESTIMATE” was used to assess the stromal score, immune score, and ESTIMATE score of tumor samples, which were correlated with the ratio of stromal and immune infiltration in the TME.

### Analysis of Clinical Characteristics

The Kaplan–Meier (K-M) method was performed to plot the survival curve, the log-rank test was used to compare the subgroups, and the Kruskal–Wallis rank-sum test was applied to analyze the association between the scores and multiple subgroups of clinical variables.

### Identification of Tumor Microenvironment–Related Differentially Expressed Genes and Enrichment Analysis

The R package “limma” was used to perform to screen Differentially Expressed Genes (DEGs) between high- and low-score groups regarding immune score and stromal score. The cutoff criteria were set as | log2 fold change (FC) | > 1 and *p* < 0.05. Then, Gene ontology (GO) functional and the Kyoto Encyclopedia of Genes and Genomes (KEGG) pathway enrichment analysis of DEGs were performed with the R packages “clusterProfiler.”

### Protein–Protein Interaction Network and Cox Regression Analysis

The STRING database (https://string-db.org) was used to construct the Protein-Protein Interaction (PPI) network encoded by DEGs, followed by reconstruction with Cytoscape software (version 3.8.2). Nodes with confidence score > 0.9 were used for building network. Univariate Cox regression was performed by using the R package “survival.”

### C-C Chemokine Receptor Type 5 Differential Expression and Survival Analyses

The Wilcoxon rank-sum test was applied to compare the difference in CCR5 expression between normal and tumor tissues. K-M analysis was performed to classify the association between the CCR5 gene and overall survival (OS).

### Gene Set Enrichment Analysis and Analysis of Gene With Clinical Characteristics

To identify biological pathways between high and low CCR5 expression groups, Gene Set Enrichment Analysis (GSEA) was performed by using GSEA v4.1.0. Then, we applied “limma” package to assess the associations between CCR5 expression and clinical characteristics.

### Analysis of Immune Cell Based on C-C Chemokine Receptor Type 5

The CIBERSORT algorithm was used to estimate the fraction of 22 tumor-infiltrating immune cells (TICs). Then, the correlation and differential analysis between CCR5 and immune cells were performed.

### Human Tumor Samples

Eight tumor tissues and corresponding peritumoral brain tissues (PBT) from LGG patients were collected in the Second Affiliated Hospital of Harbin Medical University. This research was approved by all the patients and the Ethics Committee of the hospital. Clinical characteristics of patient cohort are displayed in Supplementary Table S1.

### qRT-PCR

Total RNA was extracted from human tissues and cells by using TRIzol reagent (Invitrogen). According to manufacturer’s instructions of the Nanodrop ND-2000 spectrophotometer (Thermo Scientific™, United States), 2 μg of the total RNA was transcribed into cDNA. The SYBR Green PCR kit (Takara, Japan) was used for qRT-PCR. The relative mRNA expression was standardized by GAPDH. The primers (Tsingke Biotechnology Co., Ltd, Beijing, China) used are displayed in Supplementary Table S2.

### Western Blot

Tissues were lysed in the RIPA buffer, and protein concentration was detected with the BCA protein assay kit (Beyotime, China). The 20-μg proteins were fractionated by SDS-PAGE on 10% SDS-acrylamide gel. The membrane was blocked with 5% skim milk for 1 h. After culturing with primary (CCR5, Abcam and β-actin, Abcam) and secondary antibodies, the electro chemiluminescence kit (ECL; Solarbio, China) was used to detect the protein blots. The antibody information is listed in Supplementary Table S3.

### Immunohistochemistry

Human tissues were processed into formalin-fixed and paraffin-embedded specimens. Immunohistochemistry (IHC) was performed by means of streptavidin coupled with peroxidase. The samples were incubated overnight with the primary antibodies (CCR5, Abcam; CD4, Abcam; and CD206, Proteintech), followed by incubation with the biotinylated secondary antibody. Finally, the signals were detected using an Olympus BX41 microscope. The antibody information is listed in Supplementary Table S3.

### Statistical Analysis

All data were processed by GraphPad Prism Software (v 8.0.1, GraphPad Software, San Diego, CA, United States) and SPSS statistics software (v21.0, Chicago, United States). All measured data were expressed as mean ± SD. The comparison between the two groups used t-test. The statistical significance was established as *p* < 0.05.

## Results

### Research Pipeline

**FIGURE 1:**
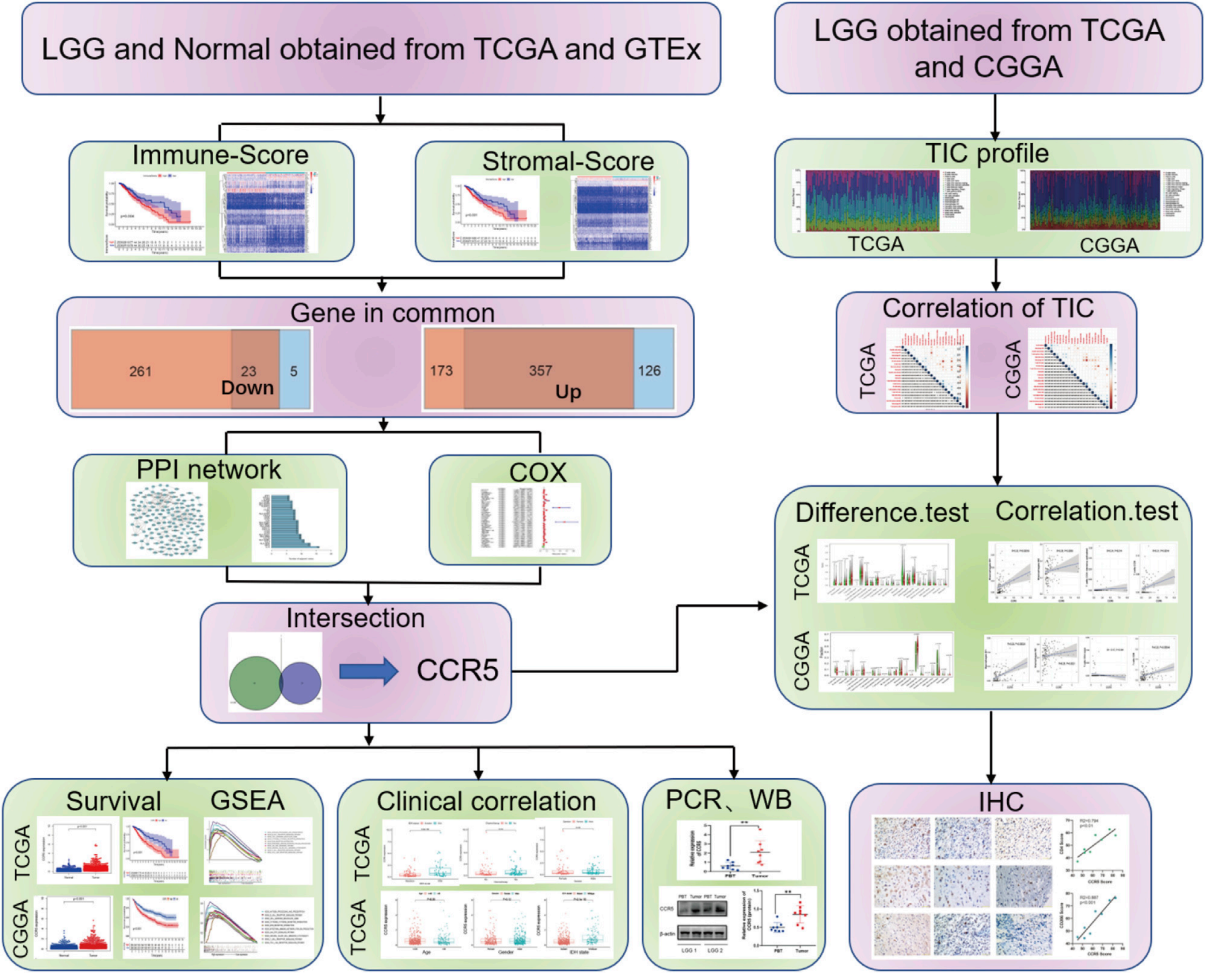
Analysis workflow of this study.

The workflow and methodology of this study is shown schematically in Figure 1. The immune score, stromal score, and ESTIMATE score based on the transcriptomic data of 529 LGG cases were calculated. DEGs identified between the low and high immune score, and stromal score groups were used to construct the PPI network and flowed into the univariate Cox regression analysis. By intersecting the hub genes in the PPI network and the significant genes in univariate Cox regression analyses, CCR5 was identified. We next showed a series of analyses of CCR5, including differential analysis, survival analysis, GSEA, clinicopathological characteristics correlation analysis, correlation with TICs, and validation in patient tissues.

### Immune Score and Stromal Score Were Correlated With Survival

**FIGURE 2:**
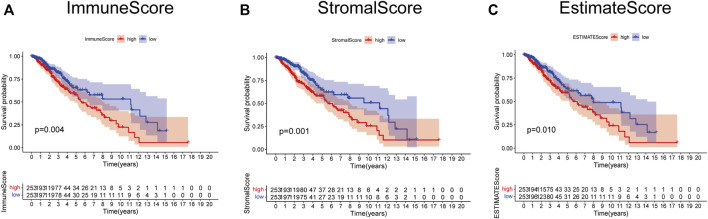
Survival analysis based on scores. Patients in the low immune score **(A)**, stromal score **(B)**, and ESTIMATE score group **(C)** had better prognosis than those in the corresponding high-score group.

The role of the TME in the progression and pathological features of several malignancies has been highlighted previously. To exhibit the significance of the TME in LGG, K-M analysis was performed to evaluate the correlation between the immune score–, stromal score–, and ESTIMATE score–based groups, and sample survival. As a result, either the immune score–high, stromal score–high, and ESTIMATE score–high group had decreased OS (Figures 2A–C). These results indicated that the fraction of immune and stromal components in the TME was negatively correlated with the prognosis of LGG patients. In addition, the immune score, stromal score, and ESTIMATE score of each LGG sample were listed in Supplementary Table S4.

### Association Between Immune Score, Stromal Score, and ESTIMATE Score With Clinicopathological Parameters of Lower-Grade Gliomas

**FIGURE 3:**
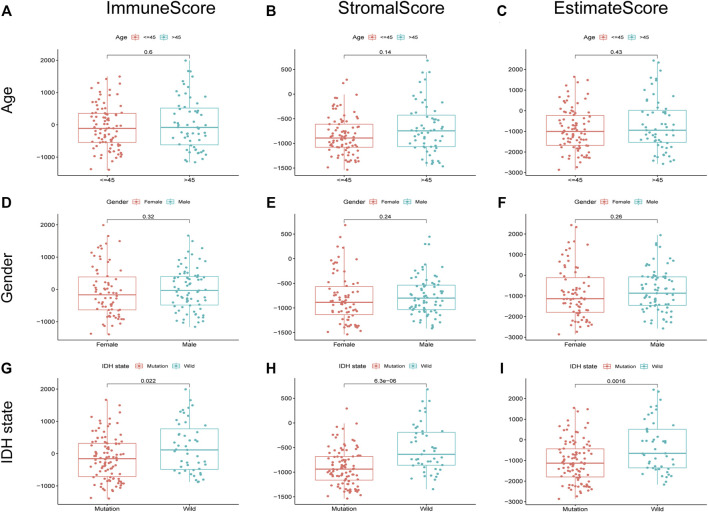
Relationship between scores and clinical characteristics of LGG. immune score, stromal score, and ESTIMATE score were not significantly correlated with age **(A–C)** and gender **(D–F)**. The IDH state was significantly associated with immune score **(G)**, *p* = 0.022), stromal score **(H)**, *p* = 6.3e-06), and ESTIMATE score **(I)**, *p* = 0.0016).

We evaluated the relationship between the proportion of immune and stromal components with the clinicopathological parameters of LGG. As a result, the correlation between the scores with age and gender was insignificant (Figure3A–F). Nevertheless, isocitrate dehydrogenase (IDH) mutation robustly predicted decreased immune score, stromal score, and ESTIMATE score, indicating that LGG with IDH mutation may have less immune and stromal infiltrations (Figures 3G–I).

### Differentially Expressed Genes Shared by Immune Score and Stromal Score Were Predominantly Involved in the Immune Response

**FIGURE 4:**
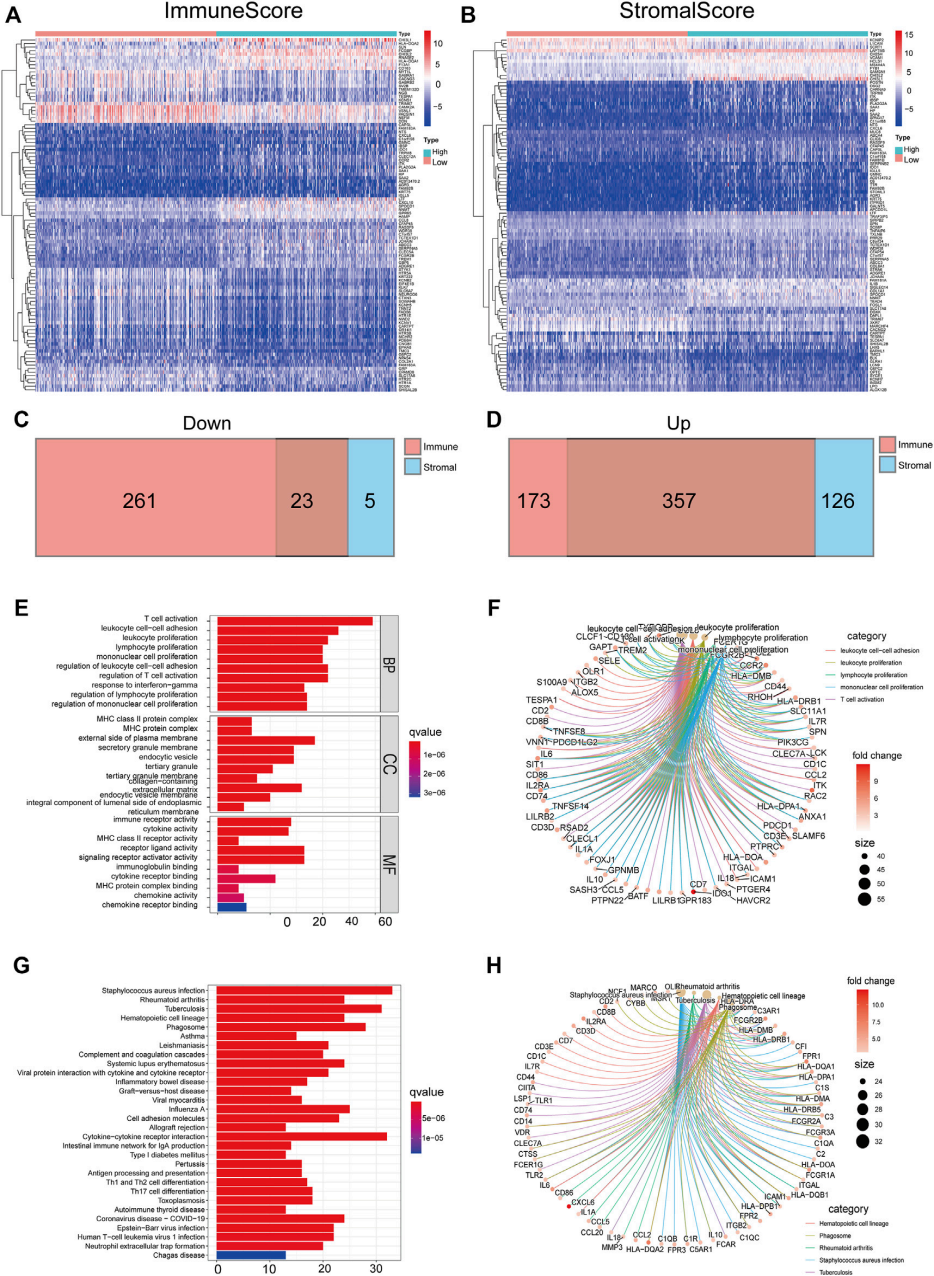
DEGs shared by immune score and stromal score were mainly immune-related genes. The heatmaps of significant DEGs between high and low immune score **(A)** or stromal score **(B)** groups. Venn plots showing common down-regulated **(C)** and upregulated **(D)** DEGs sharing immune score and stromal score, red or blue, are immune or stromal DEGs, respectively. GO-enriched functions **(E,F)** and the KEGG pathway **(G,H)** analysis of DEGs.

TME-related DEGs were screened by comparing gene expression between high-score and low-score groups regarding immune score and stromal score. Consequently, we identified 814 immune score–related DEGs including 284 down-regulated genes and 530 upregulated genes (Figure 4A). Likewise, 511 DEGs were obtained from stromal score, consisting of 28 down-regulated genes and 483 upregulated genes (Figure 4B). We also observed that 380 shared DEGs (Supplementary Table S5), among which there were 357 upregulated DEGs in immune score and stromal score (Figure 4D) and 23 down-regulated DEGs in both the groups (Figure 4C). These genes may be functionally important with regard to the status of the TME. In addition, functional enrichment analysis found that these genes were predominantly involved in the immune-related GO terms, including T-cell activation, leukocyte proliferation, and leukocyte cell–cell adhesion (Figure 4E,F), as well as KEGG pathways including Th1 and Th2 cell differentiation, chemokine signaling pathway, and IL-17 signaling pathway (Figure 4G,H). These results illustrated that shared DEGs are strongly related to immune activity, and immune factors play a crucial role in the TME of LGG patients.

### Protein–Protein Interaction Network and Univariate Cox Regression Analyses

**FIGURE 5:**
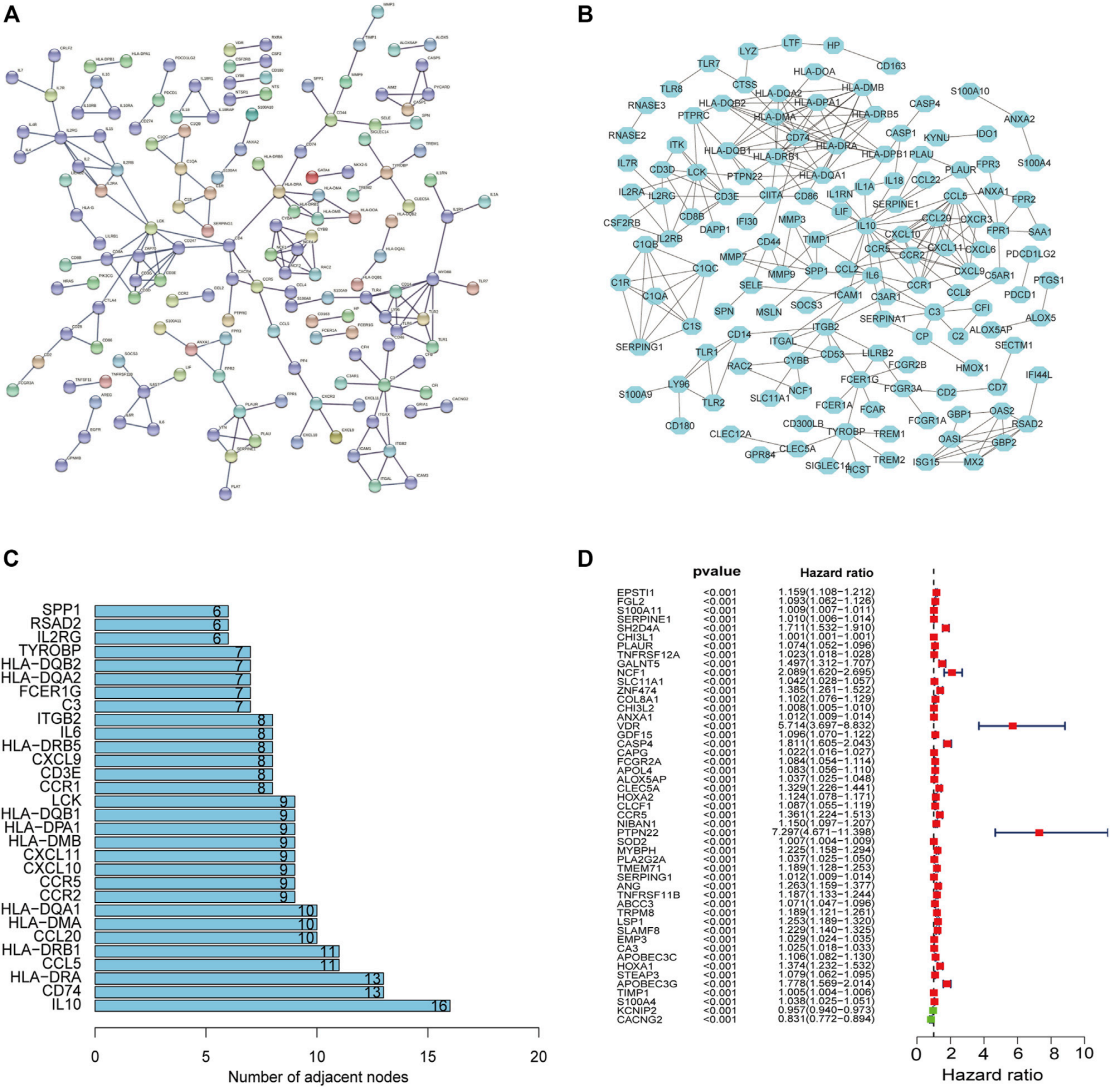
PPI network and univariate Cox regression analyses. The PPI network based on the STRING confidence score > 0.9 **(A)**. The visualization of the PPI network **(B)**. The top 30 genes ordered by the number of nodes **(C)**. Univariate Cox analyses filtered 49 genes (*p* < 0.001) related to prognosis **(D)**.

The PPI network was constructed to show the interactions among 380 DEGs based on the STRING database (confidence value > 0.9) (Figure5A). The results were visualized in Cytoscape v3.8.2 (Figure 5B), and then the bar plots were represented for the top 30 genes ranked by the number of nodes (Figure 5C), which may serve as hub nodes in the network. In addition, the 380 DEGs were subjected to univariate Cox regression analyses and 49 genes were of prognostic significance (Supplementary Table S6) (Figure5D).

### C-C Chemokine Receptor Type 5 had Potential to be an Indicator of Tumor Microenvironment Modulation

**FIGURE 6:**
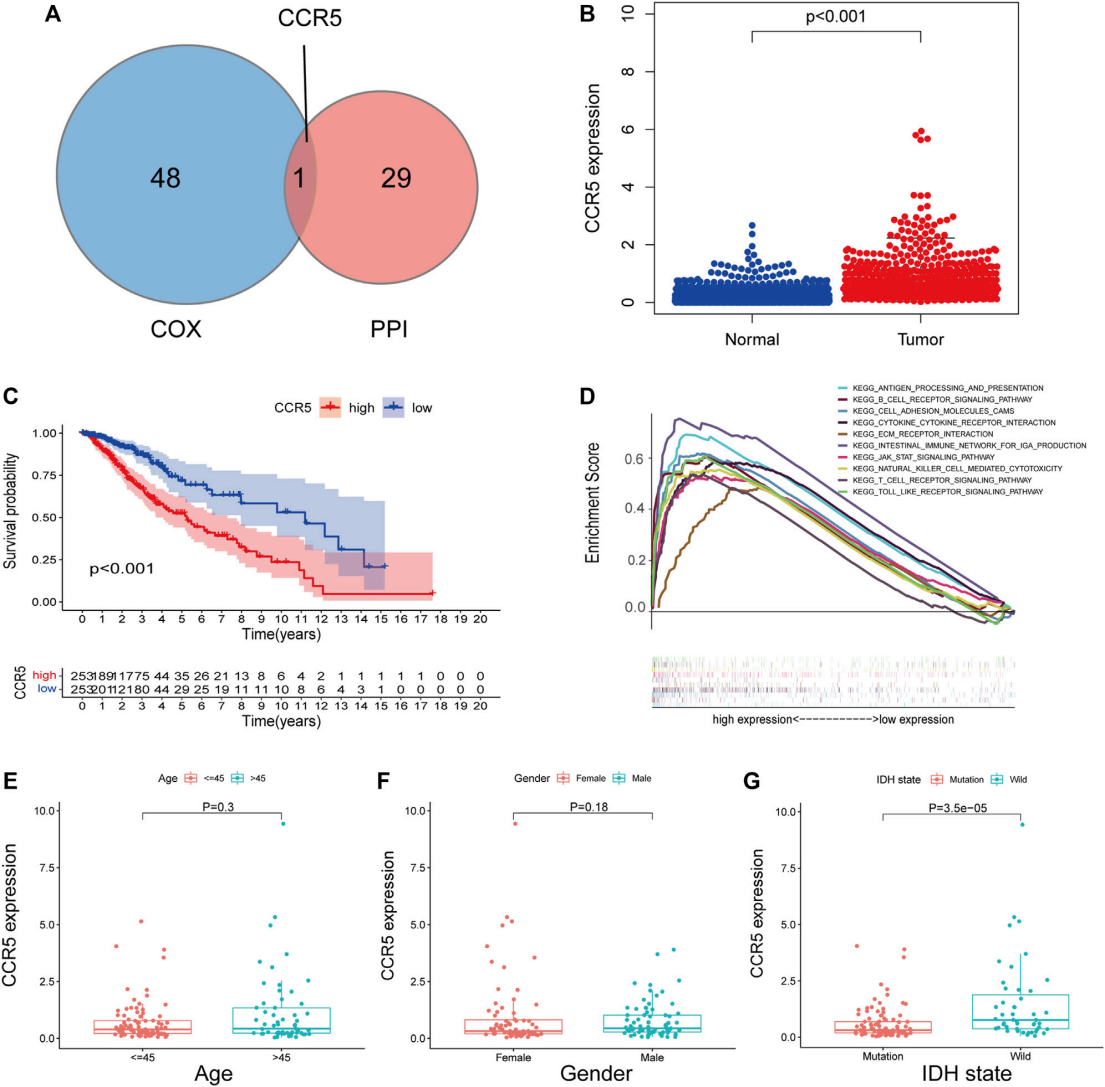
Single-gene analysis of CCR5 in the training set based on the TCGA database. The Venn plot showed CCR5 was identified based on the intersection of the 49 significant genes of Cox regression analyses and the 30 hub genes in the PPI network **(A)**. The expression of CCR5 in normal and tumor tissues of LGG **(B)**. The survival analysis of LGG patients with low and high CCR5 expression **(C)**.The GSEA analysis of CCR5 showed the top ten signaling pathways associated with the high expression of CCR5 **(D)**. The CCR5 expression was not associated with age and gender of patients **(E,F)**. The CCR5 protein expression was higher in LGG with IDH wildtype compared to IDH mutation **(G)**.

Through the intersection of 49 genes of prognostic significance with the 30 hub genes in the PPI network, CCR5 was identified (Figure 6A). Subsequently, the expression and potential role of CCR5 were explored using the TCGA and CGGA datasets, respectively. The expression of CCR5 was significantly increased in LGG comparing normal tissue (*p* < 0.001, Figure 6B; Supplementary Figure S1A). K-M analysis showed that LGG patients with decreased expression of CCR5 had prolonged survival (Figure 6C; Supplementary Figure S1B). Additionally, the top ten signaling pathways enriched in the group with the increased expression of CCR5 were exhibited (Figure 6D; Supplementary Figure S1C). Notably, these pathways were mainly associated with immune function and tumor progression, such as cell adhesion molecules cams, cytokine–cytokine receptor interaction, T-cell receptor signaling pathway, and JAK/STAT signaling pathway. Similar to the immune score and stromal score, the expression of CCR5 was increased in LGG with wildtype of IDH (Figure 6G; Supplementary Figure S1F), while it was less associated with age and gender (Figures 6E,F; Supplementary Figures S1D,E). These results suggested that CCR5 may play a role in the status of TME.

### Correlation of C-C Chemokine Receptor Type 5 With Tumor-Infiltrating Immune Cells

**FIGURE 7:**
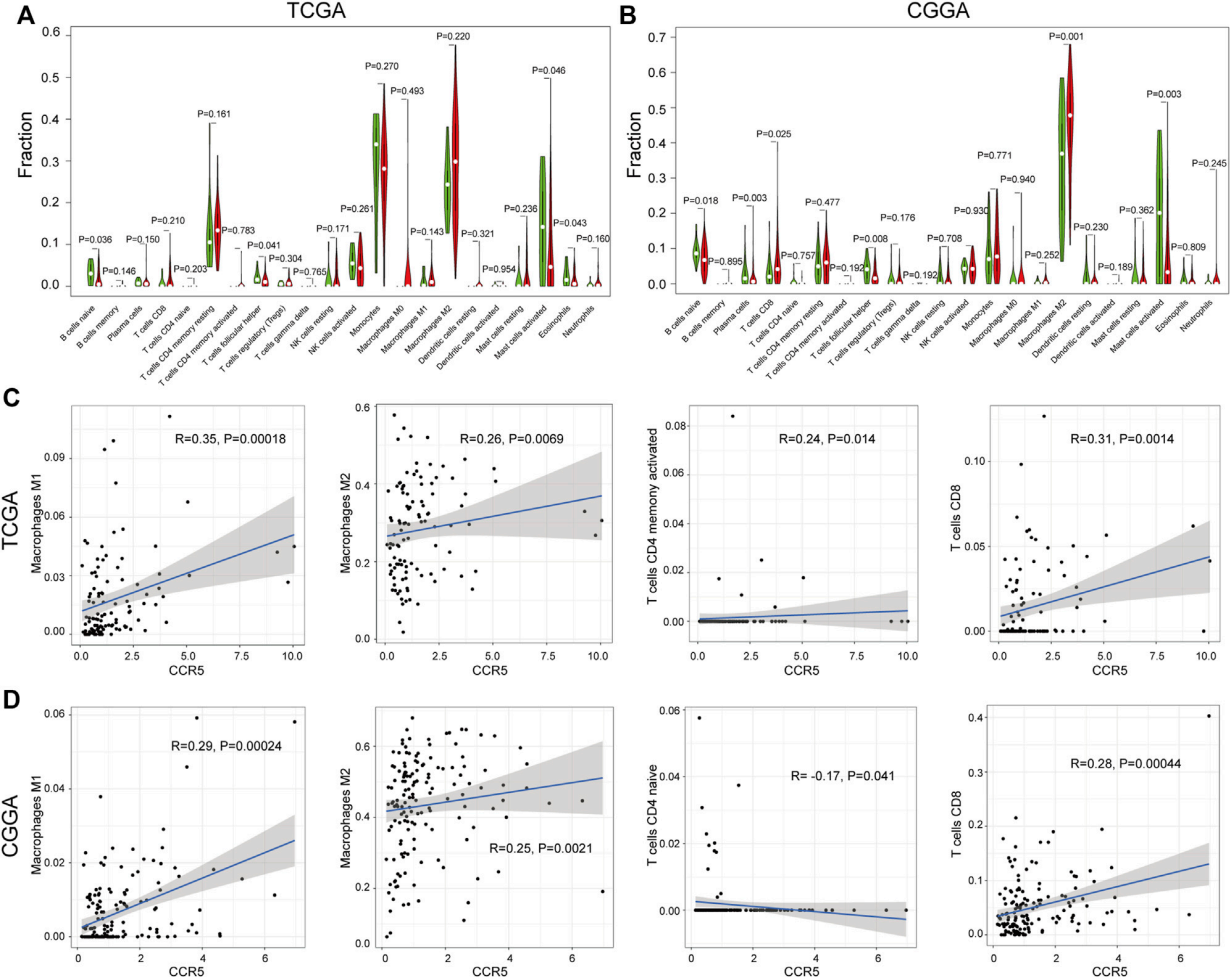
Correlation analysis between CCR5 and TICs. The difference analysis of TICs based on the expression of CCR5 in the TCGA and CGGA database, respectively **(A,B)**. The correlation analysis of CCR5 with macrophages, CD4^+^ memory-activated, and CD8^+^ T cells based on the TCGA database **(C)**. The correlation analysis of CCR5 with macrophages, CD4^+^ naive, and CD8^+^ T cells based on the CGGA database **(D)**.

To explore the correlation of CCR5 with the TIC, the fraction of tumor-infiltrating immune cells was evaluated (Supplementary Figures S2A,B). The correlation analysis of the 22 TICs in LGG was shown in Supplementary Figures S2C,D. There were significant differences in naïve B cells, T-cell follicular helpers, and mast cells activated between the CCR5-high and -low groups both in the TCGA and CGGA datasets (*p* < 0.05, Figures 7A,B). Among them, macrophages M1, macrophages M2, CD4 memory-activated T cell, and CD8 T cell are positively correlated with the expression of CCR5 (Figure7C,D). These results further corroborated our assumption that the expression of CCR5 is involved in the immune features of the TME.

### C-C Chemokine Receptor Type 5 Was Upregulated in Lower-Grade Gliomas Tissues and May Affect Immune Cell Infiltration

**FIGURE 8:**
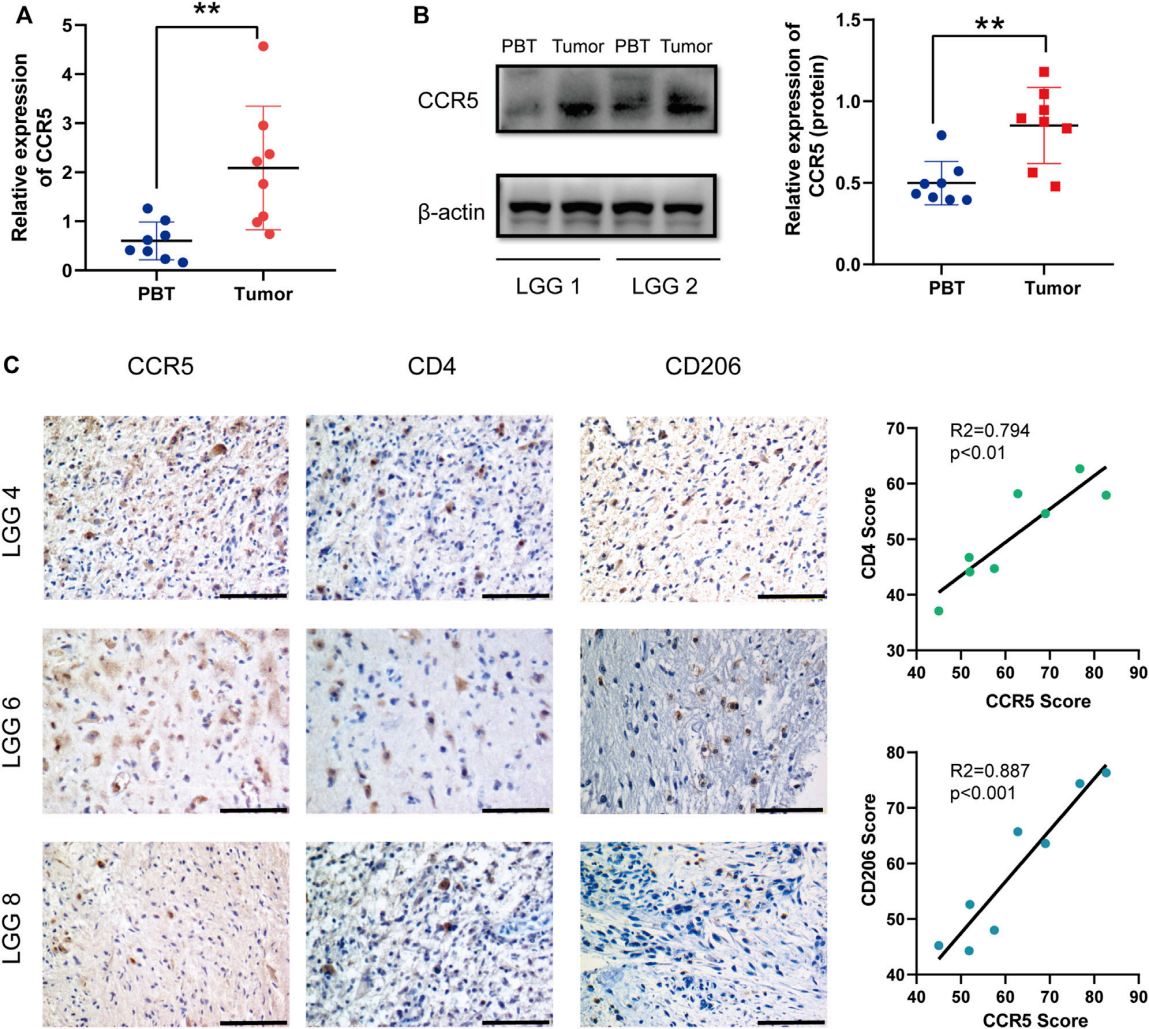
CCR5 is upregulated in LGG tissues and may affect the immune cell infiltration. qRT-PCR **(A)** and Western blot **(B)** were used to detect the expression of CCR5 in PBT and tumor of LGG patients (*n* = 8), **p < 0.01. Representative IHC staining images of the expression of CCR5 and the number of CD4^+^ T cells and M2 macrophages. The CCR5 score was positively correlated with the CD4 score and CD206 score (scale bar, 100 μM) **(C)**.

The results of qRT-PCR of 8 LGG patients showed that the mRNA of CCR5 in tumors was significantly higher than that in PBT (Figure8A). To further verify the CCR5 expression from the protein level, Western blot was also conducted based on patient tissues and further verified the significant differences of CCR5 expression between tumor and PBT (Figure 8B). Then, we performed immunohistochemical experiments on the tissues of these 8 patients to detect the distribution and expression of CCR5, CD4^+^ T cells, and macrophages. We found that the tumor of the same patient had higher CCR5 expression than PBT, and the infiltration of CD4^+^ T cells and M2 macrophages were also higher (Figure 8C).

## Discussion

It is now broadly accepted that TME contributes to progression and aggravation of glioma (Quail and Joyce 2017). Glioma obtains immunotherapeutic resistance through the reprogramming of TME (Bowman et al., 2016; Akhavan et al., 2019). In some cancers, through the binding of CCR5 to its cognate receptors, immune inhibitory signaling pathways were stimulated, resulting in an immune-suppressive state within the TME (Hemmatazad and Berger 2021). Clinical trials have recently started targeting CCR5 in the treatment of metastatic triple-negative breast cancer and colon cancer (Jiao et al., 2019). CCR5 has recently gained a lot of interest as a potential target in glioma. In the present study, we further explored the mechanism of action of CCR5 in glioma by using biological information and experimental methodology. Our findings suggest that CCR5 was associated with patient prognosis and identified to be involved in modulating the TME in LGGs.

The brain TME is composed of stromal components and various nonmalignant cells including immune cells (Oliveira et al., 2019). In recent years, TME has attracted increasing attention and plays a critical role in tumorigenesis (Jung et al., 2019; Xuan et al., 2021). TME is critical for the maintenance of cellular states in primary GBM (Pine et al., 2020). The ESTIMATE algorithm is a comprehensive algorithm for estimating tumor purity based on gene expression data (Chen et al., 2021). We calculated the immune, stromal, and ESTIMATE scores of each patient by using this algorithm. Results showed that these scores were negatively correlated with the OS rate and objectively reflected the effect of TME on the prognosis of LGG patients. Therefore, understanding the interaction between gliomas and noncancerous so-called stromal cells is crucial for the effectiveness of therapeutic strategies.

In this study, CCR5 is presented based on the intersection of the 49 significant genes of Cox regression analyses and the 30 hub genes in the PPI network. CCR5 has been confirmed to be overexpressed in a variety of tumors and affects patient prognosis (Hemmatazad and Berger 2021), such as esophageal, pancreatic, and neck cancers (Aldinucci and Colombatti 2014; Jiao et al., 2019). We confirmed experimentally and bioinformatically that the CCR5 expression was significantly higher in LGG tumor samples than in normal samples, and patients with high CCR5 expression had a worse prognosis. Furthermore, the median survival of LGG patients was correlated with IDH mutation status, and IDH wildtype LGGs have a more aggressive clinical behavior and worse outcome (Cancer Genome Atlas Research et al., 2015; Louis et al., 2016). Interestingly, our results indicated that the CCR5 expression was associated with the IDH mutation status.

The CCR5 in TME promotes tumor growth through different mechanisms (Hemmatazad and Berger 2021). It is a promiscuous receptor that binds with high affinity to CCL4, CCL5, CCL3, and CCL8; other ligands include CCL11 and CCL13 (Jiao et al., 2019). Through the binding of CCR5 to their cognate ligands, immune-activating and inhibitory signaling pathways are stimulated (Zlotnik and Yoshie 2012). The stimulation of recruitment of T regulatory cells (Tregs) and tumor-associated macrophages may promote an immunosuppressive state within the TME (Aldinucci and Colombatti 2014). Nie et al. have demonstrated that the CCR5 expression on macrophages was correlated with the M2 activation status in a mammary tumor mouse model (Nie et al., 2019). Another study found that CCR5 blockade exerts antitumor effects by promoting M1 polarization in an *in vitro* organotypic culture model (Halama et al., 2016). Based on immunohistochemistry of human LGG samples, we found that M2 macrophages labeled by CD206 were positively correlated with the expression of CCR5. The number of CD206-labeled macrophages is positively correlated with the expression of CCR5, as was CD4^+^ T cells (Figure 8C). Studies demonstrated that CCR5 enhances TGF-β–mediated killing of CD8 (+) T cells in colon cancer by recruiting Tregs (CD4 + FOXP3+) (Chang et al., 2012), and Blattner et al. reported that CCR5 induces the recruitment and homing of Tregs to the TME to stimulate immune evasion and tumor growth (Blattner et al., 2018). These observations suggest that CCR5 may affect tumor progression by mediating the infiltration of immune cells in LGGs.

**FIGURE 9:**
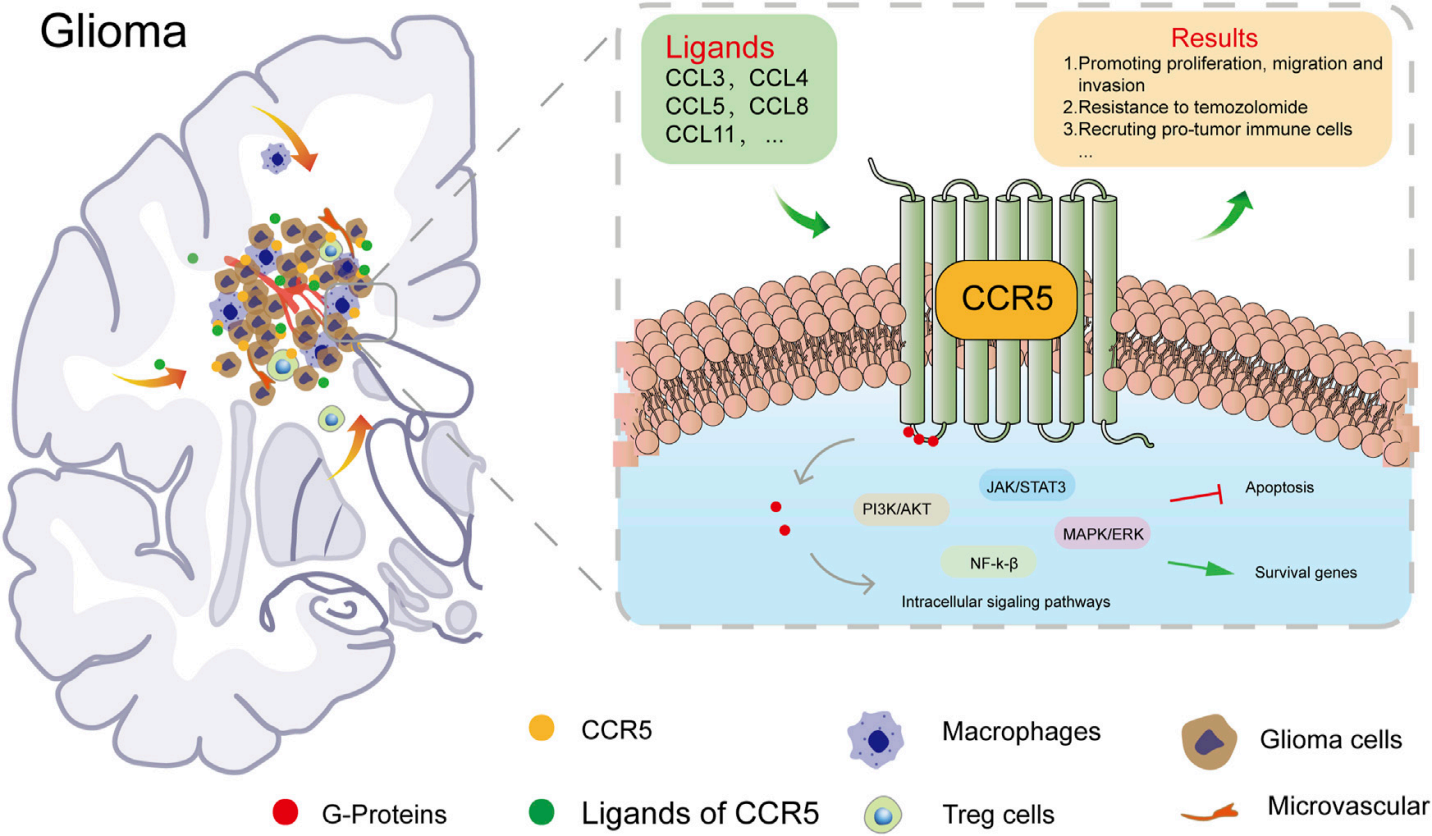
Expression and role of CCR5 in glioma.

In addition to influencing the immune infiltration of tumors, CCR5 can promote invasion and tumor cell migration by activating JAK/STAT3, MAPK/ERK, and PI3K/Akt signaling pathways (Huang et al., 2009). The GSEA analysis of CCR5 showed that cell-adhesion molecules, cytokine–cytokine receptor interaction, and JAK/STAT signaling pathway were positively associated with the CCR5 expression. Moreover, chemokines promote tumor angiogenesis by stimulating the secretion of vascular endothelial growth factor and mediating endothelial cell migration and proliferation, resulting in increased tumor cell invasion (Soria and Ben-Baruch 2008). A recent study in melanoma has convincingly demonstrated that the CCR5 expression was significantly elevated in murine melanoma cells and that CCR5 deficiency not only resulted in delayed tumor growth but also suppressed lung metastasis in mouse model (Liu et al., 2019) cancer. These findings imply that CCR5 in TME promotes tumor growth through different mechanisms. In this study, we confirmed that CCR5 could contribute to an immune-suppressive state within TME by influencing the recruitment of tumor-infiltrating immune cells such as tumor-associated macrophages in LGGs. We summarized the expression and role of CCR5 in glioma (Figure 9). Furthermore, more molecules and pathways working with CCR5 need to be deeply investigated by enrolling more experiments in our study in the future.

## Conclusion

Overall, we used the TCGA and CGGA databases to identify the TME-related gene CCR5 as a potential prognostic and immunological biomarker of low-grade glioma. Our study first pointed to the immune-modulating ability of CCR5 and its potential to regulate malignant tumor behavior in LGG, which provides a potential target for LGG in the future.

## Data Availability

The original contributions presented in the study are included in the article/Supplementary Material; further inquiries can be directed to the corresponding authors.

## Abbreviations

LGG, lower-grade glioma; GBM, glioblastomas; TME, the tumor microenvironment; CCR5, C-C motif chemokine receptor type 5; TCGA, the Cancer Genome Atlas; CGGA, Chinese Glioma Genome Atlas; GTEx, the genotype-tissue expression; KM, Kaplan–Meier; DEGs, differentially expressed genes; FC, fold change; GO, gene ontology; KEGG, Kyoto Encyclopedia of Genes and Genomes; PPI, protein–protein interaction; OS, overall survival; GSEA, gene set enrichment analysis; TICs, tumor-infiltrating immune cells; PBT, peritumoral brain tissues; IHC, immunohistochemistry; IDH, isocitrate dehydrogenase; Tregs, T regulatory cells.
