# Supplementary figures and images for: CCR5 as a Prognostic Factor in Lower-Grade Glioma is Involved in the Remodeling of the Tumor Microenvironment
Source: Front Genet. 2022 Jul 5;13:874896. doi: 10.3389/fgene.2022.874896 (PMC9294513; doi:10.3389/fgene.2022.874896)

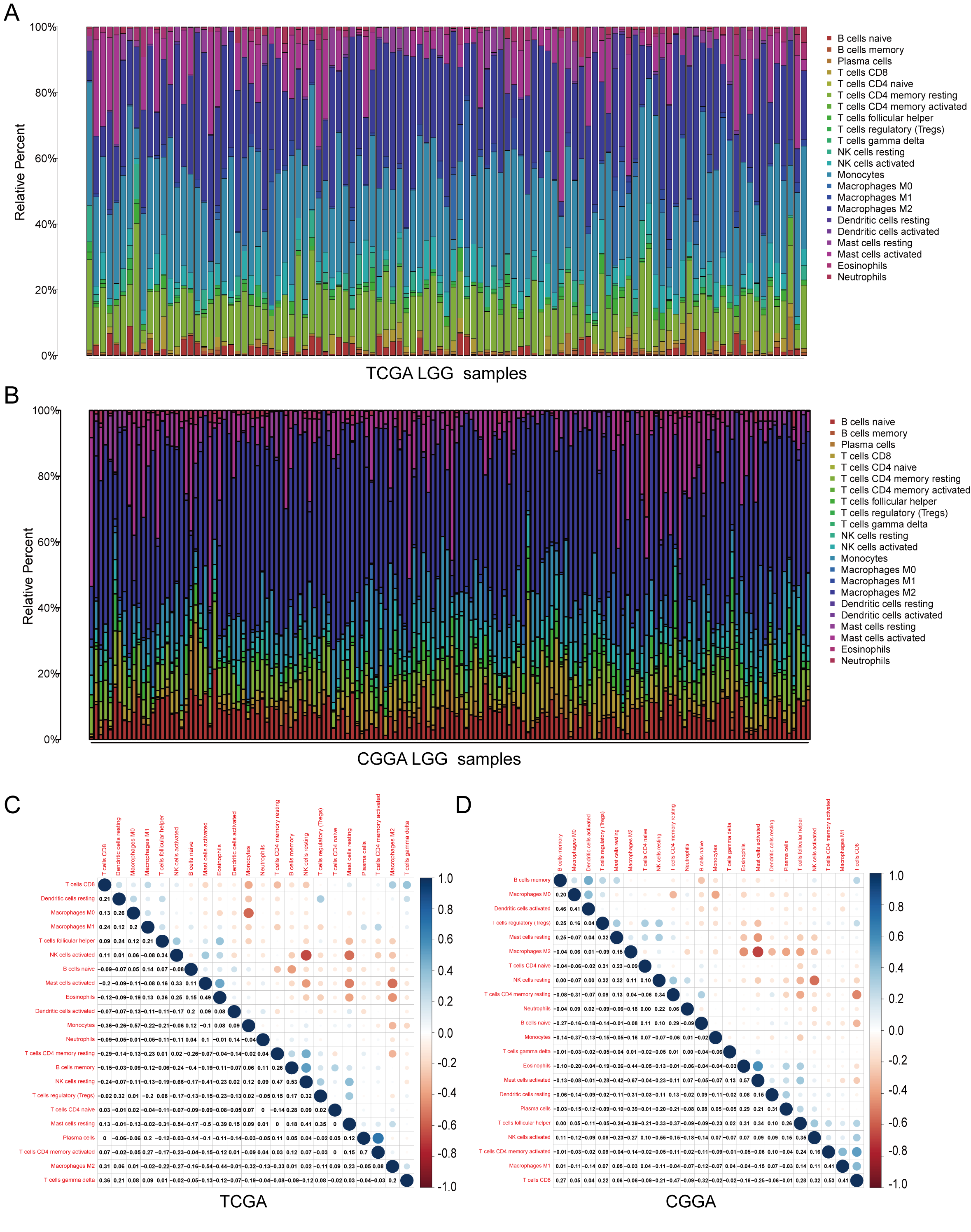

Supplement: Supplementary file 3 [file Image2.TIF]

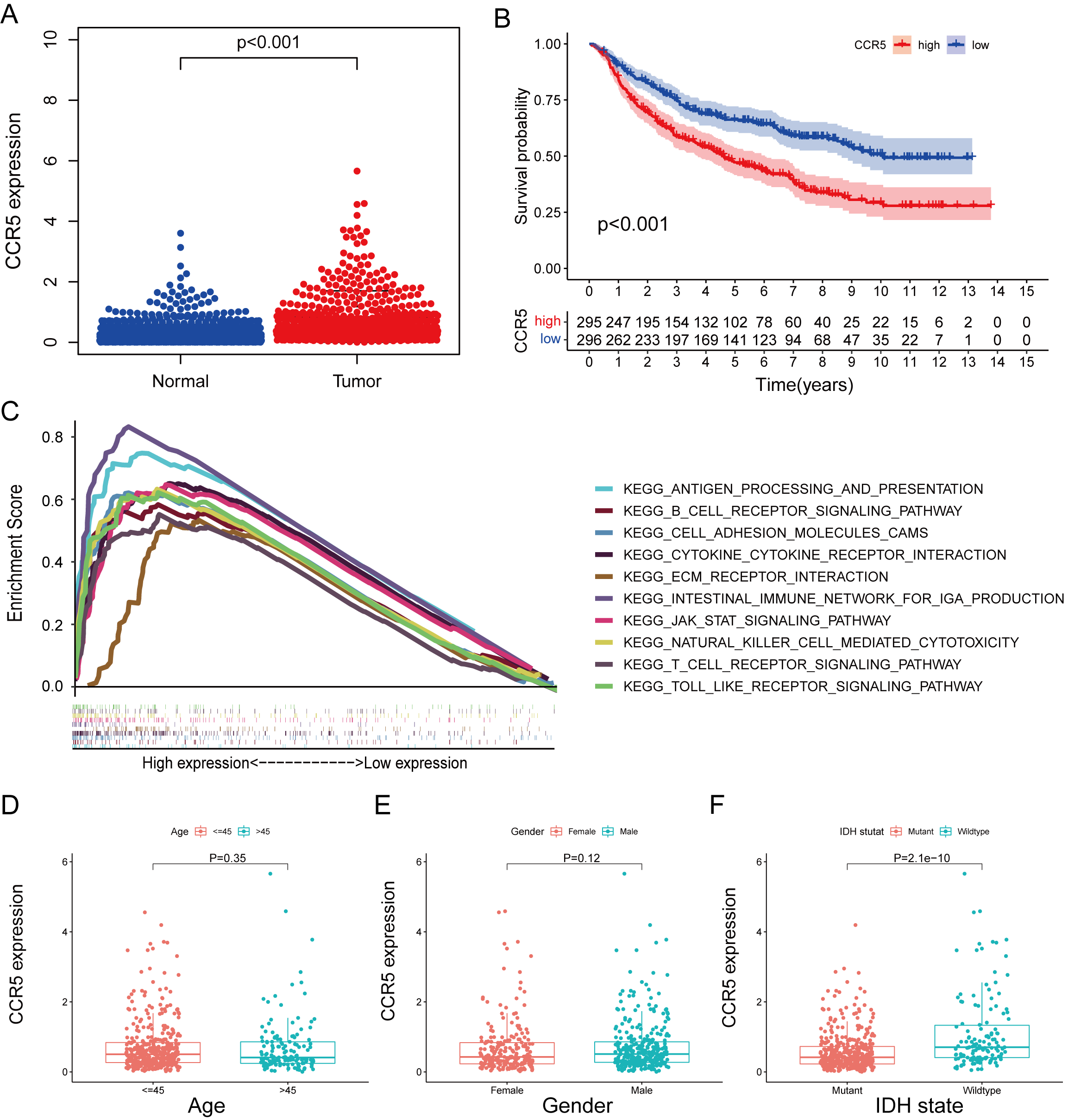

Supplement: Supplementary file 4 [file Image1.TIF]
